# Treatment of Fabry Nephropathy: A Literature Review

**DOI:** 10.3390/medicina59081478

**Published:** 2023-08-17

**Authors:** Homare Shimohata, Marina Yamashita, Kota Yamada, Kouichi Hirayama, Masaki Kobayashi

**Affiliations:** 1 Department of Nephrology, Tokyo Medical University Ibaraki Medical Center, Ami 300-0395, Ibaraki, Japank-hira@tokyo-med.ac.jp (K.H.); 2 Tsuchiura Beryl Clinic, Tsuchiura 300-0062, Ibaraki, Japan; 3 Ohba Medical Clinic, Mito 310-0841, Ibaraki, Japan

**Keywords:** chaperone therapy, enzyme replacement therapy, Fabry nephropathy, proteinuria, renal function

## Abstract

Fabry disease is an X-linked inherited lysosomal storage disorder with a deficiency of α-galactosidase A activity, which results in the intracellular accumulation of globotriaosylceramide (Gb3) and related glycosphingolipids in various organs. Fabry nephropathy is one of the major complications of Fabry disease, and kidney damage is often related to cardiovascular disease and mortality. The treatment of Fabry nephropathy thus helps prolong life expectancy. Two treatment options for Fabry nephropathy and cardiopathy are now commercially available: enzyme replacement therapy (agalsidase α agalsidase β, and a biosimilar of agalsidase β) and pharmacological chaperone therapy (migalastat). In this review, we summarize the efficacy of these treatment options for Fabry nephropathy with respect to renal function, proteinuria, and renal pathological findings. We also describe the importance of adjunctive therapy for Fabry nephropathy.

## 1. Introduction

Fabry disease is an X-linked inherited disorder caused by a deficiency of α-galactosidase A activity, which results in the intracellular accumulation of globotriaosylceramide (Gb3) and its derivatives [1,2]. The accumulation of Gb3 and globotriaosylsphingosine (lyso-Gb3, which is the deacylated form of Gb3) in various cells is responsible for the general clinical manifestations of Fabry disease, i.e., acroparesthesia, angiokeratoma, anhidrosis, hypohidrosis, cornea verticillata that appear in childhood, and renal, cardiovascular, and/or cerebrovascular complications that onset in adulthood.

The first cases of Fabry disease were reported in 1889 by Johannes Fabry and William Anderson and were described mainly as skin manifestation [3,4]. The reported prevalence of Fabry disease in 2001 was 1 in 40,000 births in Europe and the United States [5], and it is possible that the current prevalence is underestimated. Newborn screening conducted in Taiwan and Italy revealed the high frequency of newborns with lower α-galactosidase A activity: between 1 in 1000 and 1 in 3000 births [6,7]. In Japan, the prevalence of Fabry disease estimated by newborn screening was 1 in 8000 [8,9].

The phenotypes of Fabry hemizygous patients are grouped into two categories: the classical type and the later-onset type. The classical type shows general symptoms from childhood with severely reduced (<1%) residual α-galactosidase A (αGalA) activity, and the later-onset type shows cardiac, renal, or both symptoms from later ages with higher αGalA activity compared to the classical severe type. The phenotype of Fabry heterozygous female patients is also classified in two categories, but the female phenotype ranges from asymptomatic to as severe as that of male phenotype, due to random X-chromosome inactivation. αGalA gene sequencing is necessary for the definitive diagnosis of Fabry heterozygous female patients.

Fabry nephropathy is the major symptom of Fabry disease and occurs in both the classical and later-onset types. It has been reported that the mean onset age of renal manifestation(s) in Fabry disease (renal insufficiency and/or proteinuria) is 27 years, and 50% of patients with Fabry nephropathy developed end-stage renal disease by 53 years; thus, therapeutic interventions for Fabry nephropathy lead not only to favorable renal prognostic outcomes but also to satisfactory long-term life expectancy for individuals with this disease [10,11].

There are now some commercially available options for the specific treatment of Fabry disease: enzyme replacement therapy (ERT) (agalsidase α agalsidase β, and a biosimilar of agalsidase β) and pharmacological chaperone therapy (migalastat). In this review, we summarize the clinical effects and efficacy of these treatment options in Fabry nephropathy.

## 2. ERT for Fabry Nephropathy

Three formulations of recombinant human αGalA are currently available: agalsidase α (Replagal^®^), agalsidase β (Fabrazyme^®^), and a biosimilar of agalsidase β (JR-051). Agalsidase α is produced in a human cell line and is dosed at 0.2 mg/kg every other week. Agalsidase β is produced in Chinese hamster ovary (CHO) cells and is dosed at 1.0 mg/kg every other week. These two forms of enzyme replacement therapy (ERT) have identical biochemical properties and are comparable with respect to amino acid composition, but they exhibit differences in glycosylation composition and in the levels of the mannose-6-phosphate receptor (MPR)-mediated cellular uptake [12]. Lysosomal enzymes are incorporated into the kidney and heart via MPR on the plasma membrane, whereas asialoglycoprotein receptors are involved in the uptake of lysosomal enzyme in the liver [13].

According to a multicenter retrospective cohort study conducted by an independent international initiative to compare clinical outcomes of these two enzymes, there was no significant difference in the slope of the estimated glomerular filtration rate (eGFR) between agalsidase α and β treatment in patients with a baseline eGFR ≥ 60 mL or ≤60 mL, and similar rates of clinical events were observed for the two types of enzyme treatment: renal events, chronic kidney disease (CKD) G5, eGFR < 15 mL/min, renal transplantation or dialysis; cardiac events, implantation of an implantable cardioverter defibrillator (ICD) or pacemaker; cerebral events, stroke or transient ischemic attack (TIA); and death from any cause [13]. However, long-term ERT is associated with the formation of neutralizing anti-drug antibodies (ADAs) which decrease the effect of ERT by changing the catalytic activity of the enzyme and cellular uptake and accelerating decline in renal function. A significant increased risk for the formation of ADAs in male patients was demonstrated in patients treated with agalsidase-β compared to agalsidase-α. As for the influence of ADAs on renal function, Lender et al. demonstrated that a not saturated ADAs status (ADAs excess against infused enzyme dose) during infusion is associated with decreased renal function and reduced plasma lyso-Gb3 levels. The presence of ADAs was associated with a less prominent decrease in lysoGb3 following ERT in patients treated with agalsidase α, whereas the decrease in lysoGb3 after the initiation of ERT was minimally affected by the presence or absence of antibodies in patients treated with agalsidase β [14].

The effects of reducing the ERT dose and of switching from agalsidase β to agalsidase α have been investigated: in the agalsidase β dose-reduction group, the renal function decreased significantly (by ~3 mL/min) between the baseline and 1-year follow-up visit, and in the switch group, the rate of microalbuminuria was higher, with an albumin-to-creatinine ratio of 114 mg/g at baseline and 216 mg/g at the follow-up visit [15]. After this study, the same research group assessed the changes in the eGFR and the albumin-to-creatinine ratio during (*i*) treatment with the standard dose of agalsidase β, (*ii*) ERT dose reduction, and (*iii*) a subsequent switch or a direct switch to agalsidase α after 2 years of observation [16]. The eGFR values of the patients within the regular-dose group remained stable between baseline and the 2-year follow up, whereas the annual change in the eGFR was −3.74 mL/min in the dose-reduction switch groups and −2.45 mL/min in the direct switch group. The albumin-to-creatinine ratio did not differ between baseline and the 2-year follow-up in any of the three groups [16].

JR-051 is also produced in CHO cells and has a primary structure that is identical to that of the original agalsidase β, a similar charge isoform profile, and similar glycosylation profiles as well as enzyme activity [17]. In a Gla-knockout Fabry murine model, JR-051 reduced the accumulation of Gb3 in the kidney, heart, skin, liver, spleen and plasma, and there were no safety concerns regarding JR-051 in a 13-week evaluation using cynomolgus monkeys [18]. JR-051 was developed in Japan by JCR Pharmaceuticals and is available in Japan.

## 3. ERT Effects on the eGFR, Proteinuria, and Gb3 Accumulation in the Kidney

### 3.1. Agalsidase α

In the first placebo-controlled 6-month random controlled trial (RCT) including 26 male patients [19], the analysis of creatinine clearance demonstrated stable renal function with weekly agalsidase α mg/kg therapy (from baseline: 92.7 mL/min/1.73 m^2^ to after 24 weeks: 94.8 mL/min/1.73 m^2^, +2.1 mL/min, n = 13) compared to an 18% decline with placebo treatment (from baseline: 100.6 mL/min/1.73 m^2^ to after 24 weeks: 84.5 mL/min/1.73 m^2^, −16.1 mL/min, n = 11). In the analysis of inulin clearance, there was a trend in favor of the treated groups compared to the placebo group (from baseline: 77.2 mL/min/ to after 24 weeks: 71.0 mL/min, −6.2 mL/min in the treated groups vs. from baseline: 90.9 mL/min/ to after 24 weeks: 71.5 mL/min, −19.5 mL/min in placebo group). The degree of proteinuria was equal between the treatment groups and placebo group, and there was no consistent change in proteinuria in any of the groups. The degree of glycolipid inclusions assessed by toluidine blue-stained semi-thin sections in the same RCT was examined in each cellular compartment of glomeruli, tubular, and vascular and there was a decrease in glycolipid inclusions within the vascular endothelium in the treatment groups.

The subsequent open-label extension study [20] showed that the mean eGFR declined slightly after 48 months treatment with agalsidase α from 88.4 mL/min/1.73 m^2^ to 75.1 mL/min/1.73 m^2^, n = 24. A subgroup analysis revealed that the eGFR remained stable in the patients with stage Ⅰ (n = 12) or stage Ⅱ CKD (n = 8) at baseline, whereas a marked decline in the eGFR (from 47.1 mL/min/1.73 m^2^ to 24.8 mL/min/1.73 m^2^) was observed in the patients with stage Ⅲ CKD (n = 4). However, the slope of the eGFR decline in the CKD Ⅲ patients was reduced compared to that of comparable historical controls, and none of the stage III patients progressed to end-stage renal failure. The median proteinuria at the baseline was 353 mg/24 h (n = 24), and after 36 months of agalsidase α treatment the median urine protein level was 543 mg/24 h (n = 20). There was no significant change in the patients’ proteinuria during treatment, but the urine protein level of 3 of the 24 patients with overt proteinuria at baseline decreased to the level of microalbuminuria, whereas 3 of the 24 patients with microalbuminuria at baseline developed overt proteinuria during the 36-month follow-up. A pathological assessment was not conducted.

A summary of three separate prospective randomized placebo-controlled trials involving 108 adults male hemizygote patients treated with agalsidase α 0.2 mg/kg infused over a 40 min period every other day [21] is available; in these trials, the eGFR was measured using inulin, ^99^technetium DTPA, or ^51^Cr-EDTA. The averaged eGFR value at the initiation of agalsidase α therapy was 90.3 mL/min. The annualized rate of change in eGFR among 85 non-hyperfiltrating patients (eGFR > 135 mL/min) treated with agalsidase α over the next 48 months was −2.9 mL/min. Although 8 patients with baseline hyperfiltration showed large decreases in their eGFRs (−24.5 mL/min) during treatment, this result might represent the positive effect of agalsidase α therapy. The baseline mean urinary protein excretion level was 1.03 g/day, and after 1 year of agalsidase α therapy, the mean urinary protein excretion value was 0.97 g/d, and after 2 years of agalsidase α therapy, the mean urinary protein excretion was 0.97 g/d. There was no significant change in urinary protein excretion during 1 year or 2 years of agalsidase α treatment. A multivariate analysis revealed that the baseline eGFR and baseline proteinuria level (≤1 g/day or ≥1 g/day) were significant predicting factors of the eGFR decline rate.

To determine the effects of ERT with agalsidase α on renal function in patients with Fabry nephropathy, an observation study [22] was conducted by using an international registry database of patients with Fabry disease, i.e., the Fabry Outcome Survey (FOS). The mean slope change in the eGFR in all male patients (n = 115) was −2.66 mL/min/1.73 m^2^/year, with a mean change of −13.3 mL/min/1.73 m^2^ (from 94.5 mL/min to 81.2 mL/min) over 3 years of agalsidase α treatment. In all of the female patients (n = 50), the mean slope change in the eGFR was −1.2 mL/min/1.73 m^2^/year, with a mean reduction in eGFR over 3 years of −5 mL/min/1.73 m^2^ (from 71.6 mL/min to 66.6 mL/min). In a subgroup analysis, the slope change in the eGFR was −1.68 mL/min for the patients with proteinuria ≤ 500 mg/day and −3.98 mL/min among the patients with baseline proteinuria ≥ 500 mg/day. The presence or absence of hypertension at baseline did not affect the mean slope change (−2.81 mL/min vs. −2.08 mL/min). There was no significant change in proteinuria during 3 years of agalsidase α therapy, and the use or non-use of angiotensin II receptor blockers (ARBs) or angiotensin-converting-enzyme (ACE) inhibitors (ACEis) produced no obvious effect or changes in proteinuria after 3 years of ERT (males: 415 mg/day at baseline to 481 mg/dy after 3 years of ERT, females: 310 mg/day at baseline to 299 mg/day after 3 years of ERT). A renal histological analysis was not conducted.

Another 5-year observation study using the FOS [23] showed that the mean yearly reduction in the eGFR versus the baseline: after 5 years of ERT was −3.17 mL/min/1.73 m^2^ in men (n = 103) and −0.89 mL/min/1.73 m^2^ in women (n = 47). The mean yearly fall in the eGFR after 5 years of ERT was −2.83 mL/min for men and −0.87 mL/min for women with stage I CKD at baseline, −2.17 mL/min for men and −0.85 mL/min for women with stage II CKD at baseline, and −3.0 mL/min for men and −1.01 mL/min for women with stage III CKD at baseline. Symptomatic women (n = 36) who were treated with agalsidase α for 4 years were analyzed in a prospective, single-center, open-label, clinical trial [24]. The mean eGFR was 91 mL/min at baseline and 91 mL/min after 4 years of agalsidase α treatment. In the subgroup analysis of every CKD stage, an improvement in the eGFR through 4 years of treatment was observed in the stage II CKD group (n = 20). Only 1 of the 20 patients with stage II CKD and none of the 3 patients with stage III CKD demonstrated a reduction in their eGFR in excess of 5 mL/min/year while being treated with agalsidase α. The mean urinary protein level was 377 mg/day at baseline and decreased to 263 mg/day after 4 years of treatment with agalsidase α in 33 patients without corticosteroid therapy.

An observation study that extracted long-term follow up (>5 years) of FOS data [25] demonstrated that the mean yearly change in the eGFR was −2.2 mL/min/1.73 m^2^ and the mean change in the eGFR from the baseline to the year’s end was −17.6 mL/min in men (n = 134) and −4.7 mL/min in women (n = 174) during 7.4 years of agalsidase α therapy. There was no significant change in proteinuria (men: 590 mg/day at baseline to 772 mg/day at year-end, women: 331 mg/day at baseline to 420 mg/day at year-end), but a subgroup analysis of the men showed a significant increase of proteinuria in the CKD stage Ⅰ patients (eGFR > 90 mL/min) (384 mg/day at baseline to 704 mg/day at year-end). The yearly eGFR slope of the patients with proteinuria > 1 g/day was −3.9 mL/min, which was significantly worse than the eGFR slope of the patients with proteinuria < 500 mg/day and 500–1000 mg/day.

By using the FOS together with well-described cohorts of untreated individuals from Schiffmann et al.’s research [26], Beck et al. [27] determined the long-term renal outcomes of agalsidase α-treated patients. The renal outcomes (n = 268) of 5 years of ERT with agalsidase α showed that the annualized eGFR change with agalsidase α treatment was −2.86 mL/min/1.73 m^2^ among male patients with an eGFR ≤ 60 mL/min/1.73 m^2^ at baseline (n = 18) and −1.68 mL/min/1.73 m^2^ among male patients with an eGFR ≥ 60 mL/min/1.73 m^2^ at baseline (n = 117). These changes in the eGFR were smaller than those in an untreated cohort (eGFR ≤ 60 mL/min/1.73 m^2^: −6.8 mL/min/1.73 m^2^, eGFR ≥ 60 mL/min/1.73 m^2^: −3.0 mL/min/1.73 m^2^). In the same study’s female patients [27], the annualized eGFR change was 0.36 mL/min/1.73 m^2^ in the patients with an eGFR ≤ 60 mL/min/1.73 m^2^ at baseline (n = 22) and −0.43 mL/min/1.73 m^2^ in those with an eGFR ≥ 60 mL/min/1.73 m^2^ at baseline (n = 111). These changes in the eGFR were also smaller than that in an untreated cohort (eGFR ≤ 60 mL/min/1.73 m^2^: −2.1 mL/min/1.73 m^2^, eGFR ≥ 60 mL/min/1.73 m^2^: −0.9 mL/min/1.73 m^2^). Among the agalsidase α-treated patients, 57% of the males and 65% of the females had received either an ACEi or an ARB, and there was no significant difference in the annualized slope of the eGFR between patients who had not received a renin angiotensin system (RAS) inhibitor.

After the publication of these 5-year agalsidase α ERT outcome data from the FOS, the 10-year agalsidase α ERT outcome data were reported by Ramaswami et al. [28]. Their renal cohort was 152 patients, and the results of the study’s analysis demonstrated that the mean eGFR measurements at baseline and year 10 were 80.4 mL/min and 79.1 mL/min in females and 95 mL/min and 77.5 mL/min in males. The patients with an eGFR ≥ 60 mL/min/1.73 m^2^ at baseline had an eGFR slope of −0.55 for females (n = 52) and −1.99 for males (n = 79), and the patients with an eGFR < 60 mL/min/1.73 m^2^ at baseline had a mean eGFR slope of −0.14 for females (n = 10) and −2.79 for males (n = 11). In that analysis, urinary protein > 1 g/24 h was present in 3 of 45 females and 9 of 70 males at baseline, but the change of proteinuria during ERT was not investigated. Another investigation [29] revealed that the change in treatment frequency with agalsidase α from every other week to a weekly infusion improved the mean rate of change in the eGFR from −8.0 mL/min to −3.3 mL/min during the 24-month follow-up; after the switch to weekly infusions, 3 of 14 patients showed an improvement in their eGFR, and 6 of the 14 patients showed slowing in the rate of eGFR decline.

### 3.2. Agalsidase β

To evaluate the effectiveness of agalsidase β, Eng et al. [30] conducted a multicenter randomized placebo-controlled double-blind study of 58 males with Fabry’s disease who were treated with agalsidase β for 20 weeks, and they performed a subsequent 6-month open-label extension study. The patients’ eGFR did not change substantially from baseline in either the agalsidase β groups or placebo group after week 20 of the double-blind study or the subsequent 6 months of open-label treatment. That study’s primary endpoint was the percentage of patients in each group who were free of microvascular endothelial deposits of Gb3 in renal-biopsy specimens. The analysis results demonstrated that 20 of the 29 patients (69%) in the 20-week agalsidase β treatment group reached this primary endpoint, whereas none of the 29 patients in the placebo group (*p* < 0.001) did. The open-label extension study showed that 98% of the patients (42 of 43) were free of microvascular endothelial deposits of Gb3 in renal-biopsy specimens. The analysis of the effect of agalsidase β for proteinuria excretion was not performed in this study.

After the above-mentioned Phase 3 double-blind, randomized, placebo-controlled trial [31], a more detailed pathological analysis was conducted by using the same trial’s kidney biopsy data. The complete clearance of Gb3 from endothelium cells of all vasculatures as well as from the mesangial cells and interstitial cells was observed after 11 months of agalsidase β treatment. Moderate clearance was observed in smooth muscle cells, and limited clearance compared with other cell types was observed in podocytes and distal tubular epithelium. To reveal the long-term efficacy of agalsidase β, an open-label 30-month extension study [32] was conducted after the above-mentioned trial. In that study, the eGFR remained stable throughout the 30-month extension study (mean eGFR 129.5 mL/min; placebo to agalsidase β groups, 107.1 mL/min; agalsidase β to agalsidase β groups). At the baseline kidney biopsy, 6 of the 43 patients who had renal biopsy findings at the baseline demonstrated either focal glomerulosclerosis or global sclerosis in >50% of all observed glomeruli, and these patients were older with higher creatinine levels and high levels of proteinuria. The median urinary protein-to-creatinine ratio (UP/Cr ratio) was 0.221 g/gCre at baseline and remained stable at 0.198 g/gCre after 30 months of agalsidase β treatment.

Another open-label, phase III extension trial for up to an additional 54 months following a 20-week double-blind randomized placebo-controlled phase III study of agalsidase β treatment (n = 58) [33] showed that the median eGFR remained stable and in the normal range around 120 mL/min throughout the 54-month treatment period; the mean eGFR slope of 52 patients without the six rapid-progression patients was −0.4 mL/min. In a subgroup analysis, the mean rate of the decline in the eGFR for the patients with >1 g/day proteinuria at baseline (n = 10) was −7.399 mL/min, whereas the patients with <1 g/day proteinuria at baseline (n = 42) showed a mean eGFR slope of −1.005 mL/min. In the study’s histological analysis, the patients with glomerulosclerosis >50% at baseline (n = 8) showed a higher rate of eGFR decline (−8.955 mL/min/1.73 m^2^) compared to the patients with <50% glomerulosclerosis (n = 32) (−1.404 mL/min/1.73 m^2^). The median proteinuria value did not change during the course of agalsidase β treatment.

In the same extension trial [33], the complete clearance of Gb3 in renal interstitial capillary endothelial cells was achieved at 6 months (n = 49) or 54 months (n = 8) after entry into the study. At 54 months, the Gb3 clearance of glomerular endothelial cells, mesangial cells, noncapillary endothelial cells, and distal convoluted tubule/collecting duct cells was achieved with agalsidase β treatment. Moreover, long-term agalsidase β treatment decreased the Gb3 accumulation in podocytes, which heavily accumulate Gb3 due to slow turnover rates.

A randomized placebo-controlled trial of agalsidase β treatment for up to 35 months (with 51 patients in the agalsidase β treatment group and 31 patients in the placebo group) [34] demonstrated that the mean eGFR did not change between the baseline and the final assessment in the agalsidase β treatment group or placebo group; the baseline mean eGFR was 53 mL/min in treatment group and 52.4 mL/min in the placebo group. There was no significant difference in the occurrence of the primary renal endpoint, i.e., a 33% increase in the serum creatinine level from the baseline or end-stage kidney disease requiring dialysis or transplantation between the agalsidase β treatment group (n = 10 of 51) and placebo group (n = 7 of 31). The mean proteinuria value did not change significantly from the baseline to the end of the study in either group, but the results of the study’s longitudinal analysis showed a nonsignificant decrease in proteinuria for the agalsidase β group compared to the placebo group.

In a small single-center prospective open label study assessing kidney function (n = 17) [35], the patients with an eGFR ≥ 90 mL/min (n = 9) showed stale renal function (from 115 mL/min to 102 mL/min) during the 22-month observation period, whereas the renal function of the patients with an eGFR ≤ 90 mL/min deteriorated significantly from 71 mL/min to 60 mL/min. At baseline, the mean proteinuria of the patients with normal kidney function was 0.85 g/day, and the mean proteinuria of the patients with impaired kidney function was 2.3 g/day; during the treatment period, there was no significant change in proteinuria in either group.

Another observation study [36] evaluating 151 men and 61 women from the Fabry Registry who received agalsidase β for ≥2 years demonstrated that the factor making the greatest contribution to the progression of renal disease was a higher average urinary protein level and a longer time from the onset of Fabry symptom(s) to the first ERT infusion was observed in the male patients. The patients with an average UP/Cr ratio > 1 g/g had a 4.5-fold higher risk of renal disease progression compared to those with a UP/Cr ratio < 0.3 g/g, and the men who initiated treatment > 24 years after symptom onset had a 2.9-fold higher risk of renal disease progression compared to those who initiated treatment sooner.

Forty patients with advanced Fabry disease (n = 40) were evaluated in a 2013 observation study [37], and during a median follow-up of 6 years, the patients’ eGFR deceased by −2.3 mL/min/year (men: 2.4 mL/min/year, women: 1.9 mL/min/year) with agalsidase β treatment. The eGFR remained stable over time in the patients without proteinuria (<0.15 g/day) at baseline (from 88 mL/min to 88 mL/min, n = 14), whereas four of the five patients who had proteinuria > 2 g/24 h at baseline progressed to end-stage kidney disease. The eGFR in the remaining patients decreased from 48 mL/min to 27 mL/min.

In 2015, a 10-year outcome study of patients who underwent ERT (n = 52) combined with a previous phase III clinical trial, a 54-month open-label extension study, and the Fabry registry [38] was conducted to confirm the long-term effectiveness of ERT. In that study, the patients were divided into two categories: the patients with low renal involvement (LRI) and those with high renal involvement (HRI). The mean slope of the eGFR for the 32 patients in the LRI category (UP/Cr ratio < 0.5 g/g or <50% sclerotic glomeruli at baseline) was −1.89 mL/min/1.73 m^2^, and the mean slope of the eGFR for the 20 patients in the HRI category (UP/Cr ratio > 0.5 g/g or >50% sclerotic glomeruli at baseline) was −6.82 mL/min/1.73 m^2^. Moreover, the LRI patients who maintained a UP/Cr ratio < 0.5 g/g throughout the treatment showed a lower eGFR slope compared to the LRI patients whose UP/Cr ratio rose to >0.5 g/g.

The influence of baseline proteinuria was also observed in another observation study of patients who received agalsidase β treatment for ≥5 years. The annual eGFR change was −0.7 mL/min per year in the three patients with urinary protein ≤ 0.1 g/day versus −6.3 mL/min in the six patients with baseline proteinuria ≥ 0.1 g/day [39]. The most prominent difference between agalsidase α and agalsidase β is the treatment dosage. The approved dose of agalsidase β (1 mg/kg/2 weeks) is five times higher than that of agalsidase α 0.2 mg/kg/2 weeks). Tondel et al. [40] observed the complete clearance of Gb3 inclusions in the glomerular endothelial cells and mesangial cells in all young patients after 5 years of ERT, and a dose-dependent effect on the clearance of Gb3 after agalsidase treatment was observed in podocytes.

### 3.3. JR-051, a Biosimilar of Agalsidase β

A multicenter, single-arm, phase II/III clinical study (n = 16) following patients for 52 weeks was conducted to assess the efficacy and safety of the agalsidase β biosimilar JR-051 in patients with Fabry disease in Japan. [15] The eGFR values at baseline, 26 weeks, and 52 weeks were 87.3 mL/min, 93.7 mL/min, and 89 mL/min, respectively. There was no apparent change in the patients’ eGFR during the follow-up period.

## 4. Chaperon Therapy for Fabry Nephropathy

### Migalastat

The pharmacological chaperone migalastat was approved by the European Medicines Agency in 2016 and the U.S. Food Drug Administration in 2018 for the treatment of Fabry disease in patients with an eGFR > 30 mL/min and a mutation that is amenable to migalastat. It is estimated that approx. 30–50% of individuals with Fabry disease have amenable mutations to migalastat [41].

In a randomized double-blind migalastat and placebo study conducted for 6 months and a continuous open-label study of 6- to 12-month migalastat treatment, i.e., the FACETS trial [41] of Fabry patients who had mutant α-agalsidase genotype that are suitable or not suitable for migalastat therapy, there was no significant difference in the annualized change in the eGFR form baseline to month 6 (stage 1) between the migalastat group (baseline eGFR: 94.4 mL/min) and placebo group (baseline eGFR 90.6 mL/min). In the patients followed up throughout 24 months of migalastat treatment, the mean annualize changes in the eGFR from baseline to the 24-month timepoint was −0.3 mL/min. Regarding the effect of proteinuria, there were no significant differences in the baseline levels of the groups’ 24 h urinary protein excretion or in these values’ changes from baseline to the last observation period. The primary endpoint of that study [42] was the percentage of patients who achieved a response (>50% reduction in the number of Gb3 inclusions per kidney interstitial capillary at 6 months). In addition, 13 (41%) of the 32 patients who received migalastat and 9 (28%) of the 32 patients who received the placebo showed a response, with no significant difference between the groups. However, a post hoc analysis of the patients with stage I CKD and a prespecified analysis of the stage II CKD patients with a suitable mutation (n = 45) showed a significantly greater reduction in the mean number of Gb3 inclusions per kidney interstitial capillary.

The above-described randomized 18-month open-label comparison of migalastat and ERT (the ATTRACT study) [41] showed that the annualized eGFR_CKD-EPI_ from baseline to month 18 was −0.4 mL/min in the migalastat-treated group and −1.03 mL/min in the ERT group [40]. The baseline eGFR_CKD-EPI_ (using the Chronic Kidney Disease Epidemiology Collaboration [CKD-EPI] formula) was 89.6 mL/min in the migalastat group and 95.8 mL/min in the ERT group, and the two treatments had comparable effects on renal function. The mean change of 24 h urine protein was lower in the migalastat group (49.2 mg) than in the ERT group (194.5 mg). A subsequent examination of 12-month migalastat-only open-label extension period in the ATTRACT study [43] was conducted. The mean annualized rate of change in the eGFR_CKD-EPI_ from baseline (eGFR_CKD-EPI_: 90.6 mL/min) to month 30 was −1.7 mL/min in the patients who received migalastat during the ATTRACT study and continued to receive migalastat (Group 1). The mean annualized rate of change was comparable between the initial 18-month ERT treatment period (−2.0 mL/min) and the subsequent 12-month open-label period in the patients who received ERT during the ATTRACT study but discontinued the ERT and started treatment with migalastat (Group 2). No significant change from baseline in 24 h urine protein was observed in Group 1 or 2.

A post hoc analysis of the FACETS and ATTRACT trials [44] was performed to evaluate long-term changes in renal function in 78 patients with Fabry disease and an amenable mutation and in long-term open-label extension studies. During the long-term follow-ups, the patients’ renal function was generally stable irrespective of the treatment status and gender. The annualized rate of change in the eGFR_CKD-EPI_ in the ERT-naïve male and female patients was −1.8 mL/min and −1.4 mL/min and the corresponding rates in the ERT-experienced male and female patients were −2.6 mL/min and −0.8 mL/min, respectively. The mean baseline eGFR_CKD-EPI_ in the male patients was 85.8 mL/min, and that in the female patients was 93.1 mL/min.

To investigate the long-term effects of migalastat therapy in clinical practice, a prospective single-center open label study of 14 patients with Fabry disease [45] was conducted. The patients’ eGFR decreased from 76 mL/min to 72 mL/min in the therapy-naïve group during the 1-year observation period and was stable at 95.5 to 95 mL/min in the group that underwent a switch from ERT to migalastat during the same period. In addition, the single-center observational study conducted by Riccio et al. [46] on the effect of switching from ERT to migalastat in seven male patients with Fabry disease demonstrated that the patients’ renal function was stable during the migalastat treatment period (from 99.9 to 98.3 mL/min), and proteinuria was decreased after migalastat treatment (from 145 to 78.6 mg/day).

Lenders et al. [47] conducted a prospective observational multicenter study of 54 Fabry patients for 24 months of migalastat treatment under real-world conditions. As a secondary endpoint, the reduction in the eGFR from baseline to month 24 was −2.6 mL/min in the female patients (98.6 to 90.4 mL/min) and −4.4 mL/min in the males (99.8 to 92.4 mL/min). There was no significant change in albuminuria after 24 months of migalastat treatment in the females or males.

## 5. Adjunctive Therapy for Fabry Nephropathy

Maintaining proteinuria at a lower level is a rationale for CKD treatment [48], and the same is true of Fabry nephropathy. Since it is well known that ERT itself does not have an antiproteinuric effect (with the exception of one report [49]), adjunctive therapy such as the use of an ACE and/or ARB or the vitamin D analog paricalcitol is necessary for the treatment of Fabry nephropathy. An open-label, nonrandomized, prospective study conducted by Tahir et al. [50] demonstrated that ACE and/or ARB treatment in conjunction with agalsidase β (1 mg/kg body weight every 2 weeks) resulted in sustained reductions in proteinuria with a stabilization of kidney function in six patients with stage III or IV CKD [49]. Before the start of the ERT regimen, the median proteinuria in the CKD stage III, IV group was decreased from 1.24 g/day to 0.21 g/day with the use of an ACE and/or ARB; in addition, after add-on ERT, the progression rate of the eGFR was −0.23 mL/min (from 52 to 39.4 mL/min) with 30 months of follow-up in the stage III, IV CKD group.

An investigation by Warnock et al. [51] also demonstrated the effectiveness of agalsidase β combined with antiproteinuric therapy with an ACEi and/or ARB. The patients who maintained a UP/Cr ratio < 0.5 g/gCre or a 50% reduction in their baseline UP/Cr ratio by using an ACEi or ARB had a significantly better eGFR slope (−3.6 mL/min) compared to the patients who did not meet these criteria (−7.0 mL/min). As indicated by the natural history data from the Fabry registry [26] and the results of a multicenter retrospective clinical records-based study of 447 Fabry patients without ERT [52], since proteinuria has been established as a major risk factor for renal progression in Fabry nephropathy, the reduction in proteinuria with an ACEi and/or ARB is an important strategy to prevent the decrease in renal function.

Paricalcitol is another useful option to control proteinuria in Fabry nephropathy. Pisani et al. showed that add-on 1-mg paricalcitol treatment reduced proteinuria from 2.1 g/14 h to 0.4 g/day over a 6-month period in patients with Fabry nephropathy who used an ACEi and/or ARB at the maximum tolerated dosage [53]. Although the ability of paricalcitol to control proteinuria is known, transient worsening of cardiac and renal functions associated with the use of paricalcitol has been described [54]. Trimarchi et al. [55] proposed the potassium-sparing diuretic amiloride as a candidate adjuvant antiproteinuric agent in Fabry disease. Amiloride reduces proteinuria by protecting podocytes and acting on the distal nephrons.

Sodium-glucose cotransporter-2 (SGLT2) inhibitors, i.e., dapagliflozin [56] and empagliflozin [57], sacubitril-valsartan [58], and a non-steroidal mineralocorticoid receptor antagonist, finerenone [59], have exhibited excellent reno-protective effects against CKD in individuals with diabetes and have received attention as attractive treatment options for CKD. These agents might have useful potential for adjunctive therapy in Fabry nephropathy. Especially, in the DAPA-CKD study [56], not only did participants with type 2 diabetes but also participants without type 2 diabetes, including ischemic/hypertensive nephropathy, chronic glomerulonephtitis (IgA nephropathy, focal segmental glomerulosclerosis, membranous nephropathy, minimal change disease), chronic interstitial nephritis, showed a reduction in proteinuria and reno-protective effect by dapagliflozin treatment. Unfortunately, Fabry nephropathy was not included in DAPA-CKD study. However, a 2023 multicenter observational prospective cohort study [60] is going to evaluate the effect of the SGLT2 dapagliflozin in patients with both Fabry disease and CKD at stages I–III, a genetic and biochemical diagnosis of Fabry disease and albuminuria for at least 6 months despite treatment with a stable dose of ERT or Migalastat for 12 months, and ACEi or ARB titrated to the maximum tolerated dosage for at least 6 months. The observation periods are two years, and the primary object will be the effect of Dapagliflozin on albuminuria and the secondary object will be the effect of Dapagliflozin on kidney disease progression. In the future, multiple adjunctive treatment options for Fabry nephropathy combined with ERT or chaperon therapy may be established.

## 6. Conclusions

In the many reports about the treatment effects of ERT for Fabry nephropathy, the patients’ renal function remained stable during follow-up after treatment with agalsidase α, agalsidase β, or a biosimilar if these treatments were started at an early stage of Fabry disease; however, these treatments have lower efficacy in patients at a later disease stage or those with heavy proteinuria and severe renal historical damage. The clearance of Gb3 depositions in renal tissue is more dominant in patients treated with agalsidase β compared to agalsidase α, especially as sufficient clearance of Gb3 from podocyte was only observed in agalsidase β treated patents. As a chaperon therapy, migalastat exerted a reno-protective effect in early CKD-stage populations; however, it is unclear whether migalastat has the potential to prevent the deterioration of renal function in patients at later stages of CKD.

The patient’s age at the starting point of ERT or chaperon therapy, gender differences, the Fabry disease subtypes (classical or later-onset), and the presence/absence of adjunctive therapy (ACE or ARB) are important factors in evaluations of the precise effects of ERT or chaperon therapy on renal function. Since ERT did not show an anti-proteinuric effect in several studies, adjunctive therapy such as the use of a renin-angiotensin-aldosterone system (RAAS) inhibitor is essential to reduce proteinuria in patients with Fabry nephropathy. Newly emerged medicines (SGLT2 inhibitors, sacubitril-valsartan, and non-steroidal mineralocorticoid receptor antagonists) are attractive candidates for adjunctive therapy in Fabry nephropathy.

## Data Availability

Not applicable.
